# Retrospective study of incidence/prevalence of pigmentary maculopathy and retinopathy in patients receiving pentosan polysulfate sodium

**DOI:** 10.1371/journal.pone.0313497

**Published:** 2025-01-09

**Authors:** Zhong Yuan, Subusola Vaughan, Carolyn Jeffcoat, Peter Hu, Ritchie Yuson, Daniel Fife, Durga Borkar

**Affiliations:** 1 Janssen Research & Development, Global Epidemiology, Horsham, PA, United States of America; 2 Janssen Research & Development, Titusville, NJ, United States of America; 3 Janssen Research & Development, Horsham, PA, United States of America; 4 Janssen Research & Development, Statistics and Decision Science, Raritan, NJ, United States of America; 5 Johnson and Johnson Surgical Vision, Irvine, CA, United States of America; 6 Janssen Research & Development, Global Epidemiology, Titusville, PA, United States of America; 7 Verana Health, San Francisco, CA, United States of America; 8 Duke University School of Medicine, Durham, NC, United States of America; Eye Foundation Hospital / Eye Foundation Retina Institute, NIGERIA

## Abstract

**Purpose:**

To evaluate prevalence and incidence rates of pigmentary maculopathy and retinopathy (PM/PR), and visual acuity (VA) changes in patients exposed to pentosan polysulfate sodium (PPS) and in patients with interstitial cystitis (IC) not exposed to PPS.

**Methods:**

This is a retrospective cohort study (January 2015–March 2021) which included adult de-identified patients from the American Academy of Ophthalmology IRIS® Registry (Intelligent Research in Sight) and Komodo Health database. Three patient cohorts were identified: two PPS-exposed patient cohorts, and Non-PPS-exposed IC patient cohort. Key study outcomes included PM/PR/Any (defined based on prior literature regardless of PPS exposure) and PM/PR/PPS (further defined by an algorithm that was based on clinical notes and other protocol-prespecified criteria in PPS-exposed patients), and VA changes in each respective cohort.

**Results:**

Prevalence of PM/PR/Any was relatively common in patients prior to PPS exposure (4.16%–8.43%). Incidence rate of PM/PR/PPS was uncommon in both PPS-exposed cohorts (0.13–0.15 per 100 person-years). Crude incidence rates of PM/PR/Any (based on ITT analysis) varied slightly across 3 study cohorts (2.13–2.81 and 2.38 per 100-person-years for PPS-exposed cohorts and Non-PPS-exposed IC cohort, respectively). Across all 3 study cohorts, patients with PM/PR/Any appeared to have approximately 2-fold higher proportion of 3 lines of VA worsening than those without PM/PR/Any.

**Conclusion:**

Prevalence of PM/PR was common in patients prior to PPS exposure. Incidence of PM/PR/PPS that may be associated with PPS exposure was relatively uncommon. Crude incidence rates of PM/PR appeared similar across all patient cohorts regardless of PPS exposure.

## Introduction

Interstitial cystitis (IC) is a urological syndrome characterized by symptoms of urinary urgency and frequency, nocturia, and bladder pain or discomfort. The only United States (US) Food and Drug Administration (FDA)-approved oral therapy for relieving bladder pain or discomfort associated with IC is pentosan polysulfate sodium (PPS). Long-term use of PPS in clinical studies of patients with IC showed good tolerability with few or no reported serious adverse events [1–7]. However, prior studies have suggested that pigmentary maculopathy (PM) and pigmentary retinopathy (PR) could be a new safety finding linked with PPS intake [8–10].

Pearce et al. reported that funduscopic findings in patients receiving PPS showed distinctive features, including macular hyperpigmentation, orange-yellow deposits, and patchy RPE atrophy [8]. Additionally, abnormalities in the posterior pole and focal RPE thickening or elevation with associated hyper-reflectance were reported, potentially showing an association between PPS use and retinal pigmentary changes [11].

Although subsequent case series and retrospective epidemiologic studies have identified some potential risk factors and suggested a potential association between PM/PR and chronic exposure to PPS [10, 12], the totality of clinical and epidemiological evidence remains inconclusive and a direct causal relationship has not been established, as highlighted in a recent FDA review and opinion article [11] and literature review analysis [13]. In a large, commercial claims database analysis, no association was found between PPS exposure and subsequent diagnosis of maculopathy [14], in contrast to another analysis in which an association was observed [9]. It was also suggested that the PM observed in IC patients was not associated with long-term use of PPS, but rather a result of the overactive immune and inflammatory responses underlying both diseases [13, 15]. Furthermore, a retrospective, matched cohort study from a patient database reported a greater incidence and risk of retinopathy in IC patients regardless of PPS exposure [16].

In this study, we report prevalence and incidence rates of PM/PR, and changes in visual acuity (VA) in patients exposed to PPS, and in patients with IC not exposed to PPS using data from de-identified electronic health records and a medical claims database.

## Methods

### Study design

This was a non-interventional, retrospective cohort study of PPS-exposed patients and IC patients not exposed to PPS using data from American Academy of Ophthalmology IRIS^®^ Registry (Intelligent Research in Sight) and Komodo Health database. Additionally, American Urological Association Quality (AQUA) registry was explored for supporting information but was not included in the analysis.

The study period spanned from January 01, 2015-March 01, 2021, utilized all available data from IRIS Registry and Komodo database, and included adult patients ≥18 years old in 3 main cohorts: PPS Clean, PPS Overall, and Non-PPS-exposed IC. PPS Clean was defined as patients having their first documented exposure to PPS (index date) between May 22, 2018-March 01, 2021 **(S1 Fig)**, with the earlier date established based on the publication from Pearce et al. in which a potential association between PPS exposure and PM was first reported [8]. It is anticipated that the observation or medical description in medical notes regarding PM/PR in relation to PPS exposure may initially occur in clinical practice after the publication from Pearce et al. Using cross-linked databases, PPS Overall was defined as patients with first documented PPS exposure (index date) between January 01, 2015-March 01, 2021, which was intended to identify all PPS-exposed patients based on the cross-linked databases. Non-PPS-exposed IC was defined as patients with at least 1 IC diagnosis and no documented PPS exposure between January 01, 2015-March 01, 2021. This cohort was established to evaluate study endpoints in IC patients and undocumented PPS exposure to further contextualize the study endpoints observed in patients with documented PPS exposure. The Non-PPS-exposed IC cohort was not intended to conduct direct statistical comparisons. The date of first IC diagnosis during the study period was considered the index date for the corresponding cohort accordingly. There was no a priori hypothesis testing for this study, and therefore, no prespecified sample sizes were required.

This study was registered at ClinicalTrials.gov (NCT05179460) and all analyses were prespecified, unless stated otherwise. All patient data was de-identified prior to performing the analyses from September 1, 2021 to May 15, 2022. For the purposes of this study, the study was reviewed and deemed exempt by the Western Institutional Review Board-Copernicus Group Institutional Review Board (Puyallup, WA).

### Data source and variables

All variables and data were sourced from the IRIS Registry and Komodo Health database. The IRIS Registry included 70 million unique patients from over 3100 practices and ophthalmologic ICD diagnoses to evaluate key study endpoints and clinical testing for VA outcomes. Additional data were derived from the Komodo Health database which collected data from aggregated, adjudicated claims curated by one of the largest pharmacy benefits managers in the US and maintains proprietary partnerships with more than 150 key national payers (representing over 150 million payer-complete lives) and consortiums. The Komodo Health database also supplied PPS dispensing data (exposure history) through National Drug Codes, patient baseline characteristics, medication use, and IC diagnosis.

**Fig 1:**
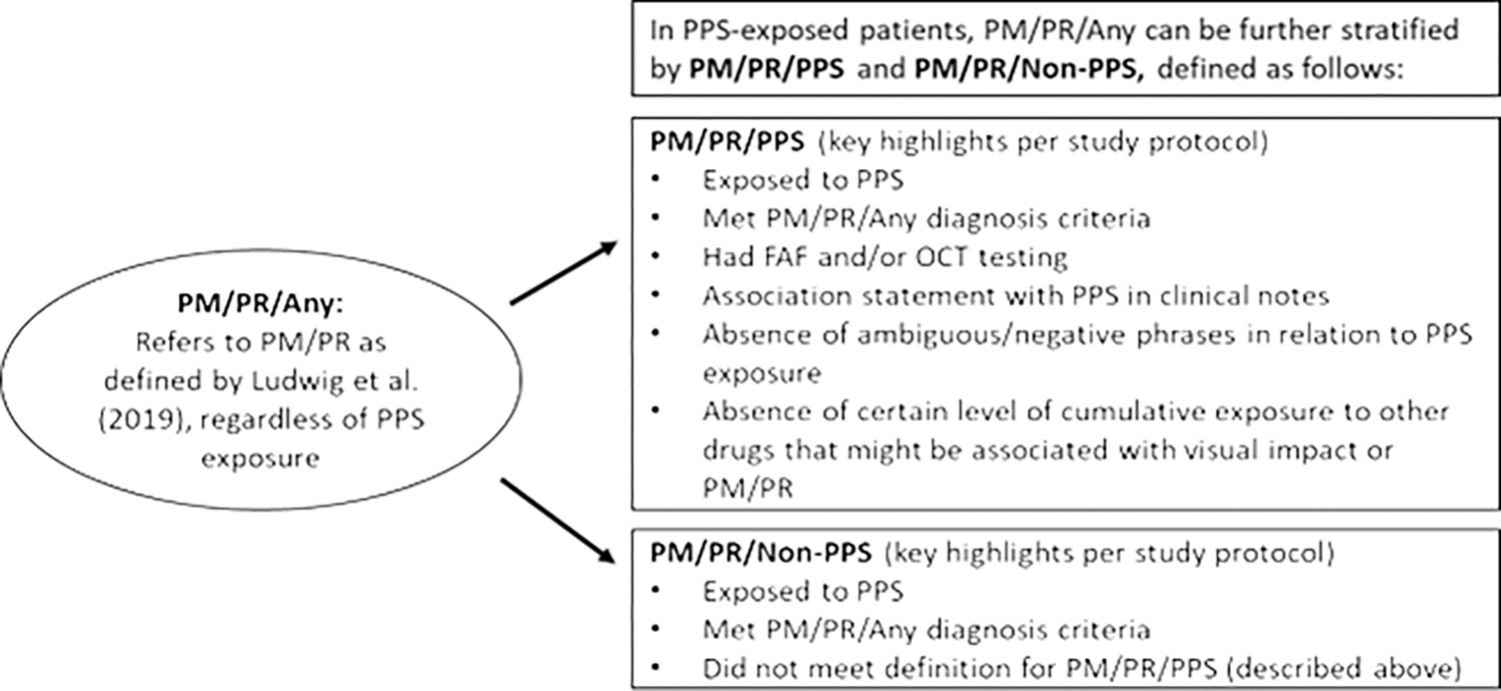
Definitions of study endpoints–PM/PR/PPS and PM/PR/Non-PPS. FAF, fundus autofluorescence; OCT, ocular coherence tomography; PPS, pentosan polysulfate sodium; PM, pigmentary maculopathy; PR, pigmentary retinopathy.

The study endpoints are PM/PR/Any, PM/PR/PPS, and PM/PR/Non-PPS, and are described in detail in the clinical study protocol (ClinicalTrials.gov Identifier, NCT05179460; Study Protocol, RWJ800077ICS4001) and Statistical Analysis Plan. PM/PR/Any refers to a medical diagnosis of PM/PR regardless of exposure to PPS, which is primarily derived from Ludwig et al. (2019) (**S1 Table**). PM/PR/PPS and PM/PR/Non-PPS refers to PPS-exposed patients and whether they met the definitions of PM/PR/PPS, respectively, and are briefly described in **Fig 1**.

The case definition for PM/PR/PPS was developed through a comprehensive feasibility assessment by the study team in collaboration with Verana Health. Cases in which macular exam or interpretation of OCT (optical coherence tomography)/FAF (fundus autofluorescence) confirmed pigmentary changes or RPE (retinal pigment epithelium) atrophy were included, and/or the assessment/plan section of the clinical notes which specifically noted “pigmentary maculopathy” associated with other keywords (“maculopathy”, “pigment changes”, “RPE changes”, “pigment clumping”, “drusen”, “atrophy”, “pigment migration”, “pigmentary retinopathy”). During feasibility testing, PM/PR cases were further assessed for whether the clinician suspected the maculopathy was related to PPS and was confirmed by a board-certified retina specialist reviewing the records (keywords included “PPS toxicity”, “toxic maculopathy due to PPS,” “pigmentary maculopathy likely related to PPS”). Further details are provided in the study protocol. PM/PR/PPS events accounted for only a small proportion of PM/PR/Any cases, thus results for PM/PR/Non-PPS are not described in this article because they were largely redundant to the results of PM/PR/Any.

VA progression analyses were limited to patients who had ≤2 VA measurements in the IRIS Registry. VA endpoints were presented as best documented VA. Because correction is not needed in patients with 20/20 vision (or better), VA was assumed to be best-corrected for patients whose VA was 20/20 (or better) at a specific visit but whose VA was not specified as best-corrected. VA conversion methods, including conversion of Snellen VA to logMAR units and letters, followed previous literature [17, 18].

### Statistical analysis

For descriptive statistics, continuous variables were summarized using mean (± standard deviation [SD]) and median (interquartile ratio [IQR]). Counts and proportions were used to summarize categorical variables. Prevalence and incidence rates, and incidence proportion of study endpoints (PM/PR/Any, PM/PR/PPS) were calculated for PPS Clean and PPS Overall cohorts.

Any diagnosis of the study outcomes (PM/PR/Any, PM/PR/PPS) that was observed on or prior to the index date was considered a prevalent case, while those observed only on or after the index date were considered an incident case unless a same type of prevalent event was observed in the same eye. If laterality information was not available (ICD 9/10 codes), patient level information was used to determine incident cases. In the Non-PPS-exposed IC Cohort, the index date for this cohort was date of first IC diagnosis during the study period and incidence rate for PM/PR/Any was calculated.

For incidence rate calculations, on-treatment time-at-risk was defined as total duration of persistent therapeutic time from index date (first date of PPS dispensing) to earliest of occurrence of study endpoint, end of inferred persistent exposure (allowing 60-day gap between dispensing); the last day of observation of the linked database, or end of study period. Intent-to-treat (ITT) time-at-risk was defined as total duration of follow-up time from index date (first date of PPS dispensing) to earliest occurrence of study endpoint, last day of the linked database, or end of study period. Incidence rates stratified by subgroups based on age, sex, or race were also calculated.

95% CIs for prevalence rate and incidence proportion calculations used normal approximation, or Fisher’s exact test when the number of study outcomes of interest were <5. Otherwise, incidence rates, 95% CIs were calculated based on assumption of the Poisson distribution. VA changes were defined as described below and reported as a proportion for the 3 main cohorts (PPS Clean, PPS Overall, and Non-PPS-exposed IC) from baseline to last VA measurement in the IRIS Registry: no change (refers to <1 line of worsening or improvement), 1 to <3 lines of worsening, ≥3 lines of worsening, 1 to <3 lines of improvement, or ≥3 lines of improvement, respectively.

## Results

### Baseline characteristics

A total of 27,958,962 patients with records were retrieved from both the IRIS Registry and Komodo Health database from January 01, 2015-March 01, 2021. After applying selection criteria, the PPS Clean cohort comprised 3,632 eligible patients **(Table 1)**. Similarly, cross-linked databases between the IRIS Registry and Komodo were also used to identify patients for the PPS Overall (14,053 eligible patients) and Non-PPS-exposed IC (48,564 eligible patients) cohorts **(Table 1)**. Demographic characteristics were similar between PPS Overall and PPS Clean cohorts **(S2 Table)**. In both PPS-exposed cohorts, mean age was 56 years and females (88%) comprised most of all patients. Most patients were White or Caucasian (68%), followed by unknown race (23%) and Black or African American (4%). For the IC Non-PPS-exposed cohort **(S2 Table)**, mean age was 57 years, and most patients were female (90%), White or Caucasian (70%), followed by unknown race (22%) and Black or African American (5%). The specialty of the treating providers was similar across both PPS Overall and Clean cohorts, respectively (**S3 Table**). Although the ages and sexes were similar, the Clean cohort had a higher proportion of IC patients at baseline compared with the PPS Overall cohort (58% versus 46%, respectively). Comorbidities, including general, urologic, autoimmune, and ocular comorbidities prior to index date were also higher in the PPS Clean cohort compared with PPS Overall cohort, although it is worth noting that PPS Clean cohort had a relatively longer baseline period compared with PPS Overall and Non-PPS-exposed IC cohorts due to the database cutoffs. Medication use associated with the possibility of developing crystalline maculopathy (tamoxifen, canthaxanthine, talc, methoxyflurane, ritonavir, nitrofurantoin, and fludarabine) and optic neuropathy (amiodarone, ethambutol, phosphodiesterase type 5 inhibitors, and tetracyclines) was consistently observed for either baseline or concomitant therapies across the PPS Overall, PPS Clean, and Non-PPS-exposed IC cohorts **(S2 Table)**.

**Table 1:** Selection criteria for study cohorts.

	PPS Clean	PPS Overall	Non-PPS-exposed IC
Database populations	N	N	N
Total IRIS Registry Patients (Jan 01, 2015 to Mar 01, 2021	61,739,749	61,739,749	61,739,749
Total Komodo Health patients (Jan 01, 2015 to Mar 01, 2021)	32,681,108	32,681,108	32,681,108
**Inclusion criteria**	**N** ^**1**^	**N** ^**1**^	**N** ^**1**^
Presence in both the IRIS Registry and Komodo Health database (Jan 01, 2015 to Mar 01, 2021; pharmacy coverage)	27,958,962	27,958,962	-
Presence in both the IRIS Registry and Komodo Health database (Jan 01, 2015 to Mar 01 2021; medical coverage)	-	-	24,973,116
Record of their first PPS dispensing record in Komodo Health database (May 22, 2018 to Mar 01, 2021)	4,560 (100%)	-	-
Record of at least 1 PPS dispensing record in Komodo Health database (Jan 01, 2015 to Mar 01, 2021)	-	18,109 (100%)	-
Record of at least 1 diagnosis of IC in Komodo Health database (Jan 01, 2015 to Mar 01, 2021)	-	-	69,171 (100%)
Have at least 6 months of eligibility in Komodo Health database prior to index date	3,648 (80%)	14,113 (77.93%)	57,912 (83.72%)
**Exclusion criteria**	**N** ^**1**^	**N** ^**1**^	**N** ^**1**^
Patients with missing or “not reported” age or sex data	3,648 (80%)	14,113 (77.93%)	48,773 (70.51%)
Patients less than 18 years of age at index	3,632 (79.65%)	14,053 (77.6%)	48,773 (70.51%)
**Final study population**	3,632 (79.65%)	14,053 (77.6%)	48,546 (70.18%)

^1^The number of eligible patients after applying inclusion and exclusion criteria accordingly.

IC, interstitial cystitis; IRIS, Intelligent Research in Sight N, number; PPS, pentosan polysulfate sodium.

### Incidence and prevalence for the main cohorts

In the PPS Clean (N = 3,632) and PPS Overall (N = 14,053) cohorts, 306 and 584 prevalent cases, respectively, met the criteria for PM/PR/Any. The prevalence rate of PM/PR/Any in the PPS Clean and PPS Overall cohorts was 8.43% (95% CI: 7.52%, 9.33%) and 4.16% (95% CI: 3.83%, 4.49%), respectively **(Table 2)**.

**Table 2:** Prevalence of study endpoint among PPS-exposed patients.

	Count of patients with endpoint	Prevalence rate^3^ (95% CI)
**PPS clean cohort** ^**1**^		
PM/PR/Any	306	8.43 (7.52, 9.33)
PM/PR/PPS (OCT or FAF)	0	0
PM/PR/PPS (OCT and FAF)	0	0
**PPS overall cohort** ^**2**^		
PM/PR/Any	584	4.16 (3.83, 4.49)
PM/PR/PPS (OCT and/or FAF)	2	0.014 (0.0, 0.034)
PM/PR/PPS (OCT and FAF)	1	0.007 (0.0, 0.021)

FAF, fundus autofluorescence; OCT, optical coherence tomography; PM, pigmentary maculopathy; PPS, pentosan polysulfate sodium; PR, pigmentary retinopathy.

^1^Prevalence of study endpoints (PM/PR/Any, PM/PR/PPS) prior to the index date, among PPS-exposed patients since May 22, 2018.

^2^Prevalence of study endpoints (PM/PR/Any, PM/PR/PPS) prior to the index date, among PPS-exposed patients since January 01, 2015.

^3^Any diagnosis of the study outcomes (PM/PR/Any, PM/PR/PPS) that was observed on and prior to the index date was considered a prevalent case; any diagnosis of the study outcomes (PM/PR/Any, PM/PR/PPS) that was observed after the index date was considered an incident case, unless a same type of prevalent event was observed in the same eye. If laterality information was not available (ICD 9/10 codes), patient level information was used to determine if the cases would be considered incidence cases.

In the PPS Clean cohort, crude incidence rate (per 100 person-years) was 2.13 (95% CI: 1.72, 2.55) for PM/PR/Any and 0.13 (95% CI: 0.03, 0.23) for PM/PR/PPS based on ITT analysis **(Table 3A)**. These estimates were alike between patients with cumulative exposure to PPS <500 g and all PPS-exposed patients; only 2 patients in the PPS Clean cohort had cumulative exposure to PPS ≥500 g and neither of them were determined to have PM/PR/PPS. In the PPS Overall cohort, incidence rate (per 100 person-years) was 2.81 (95% CI: 2.66, 2.96) for PM/PR/Any and 0.15 (95% CI: 0.12, 0.19) for PM/PR/PPS based on the ITT analysis **(Table 3A)**. In the Non-PPS-exposed IC cohort, the incidence of PM/PR/Any per 100 person-years was 2.38 (95% CI: 2.30, 2.46) **(Table 3B)**.

**Table 3:** Incidence of Study Endpoints Among (A) All PPS-exposed and (B) Non-PPS-exposed IC patients.

**A.**								
				**On-Treatment**			**ITT Analysis**	
	**Total persons at risk**	**Count of patients with endpoint ITT**	**Incident proportion (95% CI) (ITT)**	**Count of patients with endpoint OTT**	**Incidence rate (per 100 person-years)**	**(95% CI)**	**Incidence rate (per 100 person-years)**	**(95% CI)**
**PPS clean cohort** ^**1**^								
PM/PR/Any	3,331	102	3.06 (2.48, 3.65)	82	2.07	(1.67, 2.47)	2.13	(1.72, 2.55)
PM/PR/PPS (OCT and/or FAF)	3,632	7	0.19 (0.05, 0.34)	4	0.09	(0.0, 0.18)	0.13	(0.03, 0.23)
PM/PR/PPS (OCT and FAF)	3,632	3	0.08 (0.0, 0.18)	2	0.05	(0.0, 0.11)	0.06	(0.0, 0.12)
**PPS overall cohort** ^**2**^								
PM/PR/Any	13,486	1,288	9.55 (9.05, 10.05)	838	2.83	(2.67, 2.98)	2.81	(2.66, 2.96)
PM/PR/PPS (OCT and/or FAF)	14,051	76	0.54 (0.42, 0.66)	37	0.11	(0.09, 0.15)	0.15	(0.12, 0.19)
PM/PR/PPS (OCT and FAF)	14,052	58	0.41 (0.31, 0.52)	29	0.09	(0.057, 0.12)	0.12	(0.09, 0.15)
**B.**								
**N = 45,930**	**Count of patients with endpoint**		**Incident proportion (95% CI) (ITT)**		**Incidence rate (ITT) (per 100 person-years)**		**(95% CI)**	
PM/PR/Any	3,228		7.03 (6.79, 7.26)		2.38		(2.30, 2.46)	

CI, confidence interval; FAF, fundus autofluorescence; ITT, Intent-to-Treat; OCT, ocular coherence tomography; OTT, On-treatment Time; PM, pigmentary maculopathy; PPS, pentosan polysulfate sodium; PR, pigmentary retinopathy.

^1^Incidence of PM/PR/Any and PM/PR/PPS among all PPS-exposed patients at risk since May 22, 2018.

^2^Incidence of PM/PR/Any and PM/PR/PPS among all PPS-exposed patients at risk since January 01, 2015.

CI, confidence interval; ITT, Intent-to-Treat; PM, pigmentary maculopathy; PPS, pentosan polysulfate sodium; PR, pigmentary retinopathy.

### Incidence and prevalence by subgroups

In the PPS exposed cohorts, when stratified by age, the highest prevalence of PM/PR/Any was observed in patients >70 years old (PPS Clean, 22.29%; PPS Overall; 12.88%) **(S4 and S6 Tables)**. Males also had a higher rate of PM/PR/Any than females (11.69% versus 7.93%) for PPS Clean cohort, with a similar pattern observed for PPS Overall cohort (6.01% versus 3.91%) **(S4 and S6 Tables)**.

Higher incidence rate of PM/PR/Any (per 100 person-years, ITT) in the PPS Clean cohort was observed in the older age groups (60–69 years old, 3.68; >70 years old, 3.91) **(S5 Table)**. These patterns were also generally consistent for the older age groups (ages ≥70) in the PPS Overall and the Non-PPS-exposed IC cohorts (6.67 and 6.11, respectively) **(S7 and S8 Tables)**. The incidence rate (per 100 person-years) of PM/PR/Any was relatively similar between females and males for PPS Clean cohort (2.22 vs. 1.52), PPS Overall cohort (2.88 vs 2.27), and Non-PPS-exposed IC cohort (2.33 vs 2.84) **(S5–S8 Tables)**. Sample sizes were generally too limited for most race subcategories to make meaningful interpretations across different cohorts.

### Changes in visual acuity

For VA changes, a general pattern was noted across all 3 cohorts (PPS Clean, PPS Overall, and Non-PPS-exposed IC cohorts). For the PPS Clean and Overall cohorts, among patients without PM/PR/Any, approximately half of patients did not experience VA changes during the study period. However, VA was more likely to generally worsen over time for patients with PM/PR/Any **(S9 and S10 Tables)**. There was a general pattern that the proportion of patients with ≥3 lines of VA worsening appeared higher in patients with PM/PR/Any versus those without PM/PR/Any (PPS Clean, 9.03% vs. 4.55%; PPS Overall, 12.00% vs. 4.68%; Non-PPS-exposed IC, 10.30% vs. 4.59%) **(S9 and S10 Tables)**. Because PM/PR/PPS accounted for only a very small fraction of PM/PR/Any cases, the results of VA changes were not summarized for patients with PM/PR/PPS due to limited sample size.

## Discussion

Previous case series and retrospective epidemiologic studies have suggested an association between PM/PR and chronic treatment exposure to PPS, although a direct causal relationship has not been established and results have been inconclusive as to whether PPS exposure is associated with development of PM/PR [8–11, 13, 14]. This is the first retrospective study using cross-linked de-identified medical records from the IRIS Registry and Komodo claims database to evaluate prevalence and incidence rates of study endpoints and VA changes in PPS-exposed patients (PPS Clean and PPS Overall cohorts) and in Non-PPS IC patients. The condition of PM/PR encompasses a spectrum of ophthalmologic abnormalities, with age-related macular degeneration (AMD) being the most common and seen in this study. Many modifiable (including smoking, body mass index, and cardiovascular disease) and non-modifiable (including age, family history or genetic disorder, and refractive error) factors may play a role in the development of the condition, in which age, cigarette smoking, and family history have been consistently demonstrated in some epidemiologic studies [19]. Despite PPS being the only FDA-approved oral medication commonly used for relieving bladder pain or discomfort associated with IC, the potential association between retinal pigmentary changes and PPS exposure had not been described until a case series reported by Pearce et al. (2018) [8], followed by several other published reports [10, 12, 13, 16, 20, 21].

However, as highlighted in a recent FDA review and opinion article [11], evidence remains inconclusive as to whether such retinal abnormalities are a result of PPS exposure. In addition, a comprehensive literature review reported that PPS exposure is not a causative factor of the PM observed in IC patients, and suggested that the contributing factor is due to the underlying inflammatory state common to both PM and IC [13]. Maculopathy or retinopathy is commonly diagnosed and managed by retinal specialists who utilize multimodal imaging including (but not limited to) FAF and OCT testing, and who have increased familiarity with maculopathies, including pattern dystrophies. In this study, it was noted that approximately half of the patients received their care from optometrists and general ophthalmologists, reflecting the specialty designated for the provider at the time of medical encounter. Furthermore, there are no specific ICD codes for a clinical diagnosis documenting these changes, which poses challenges in designing a comprehensive epidemiologic study [16]. The definitions used for PM/PR/PPS are also not intended to draw any causal relationship between PM/PR diagnosis and PPS exposure, but rather to reflect on how these patients are captured in the databases based on current clinical practice. It is possible that some PM/PR/Any cases observed in the PPS Cohorts or in the Non-PPS-exposed IC cohort may have similar clinical presentation as the PM/PR/PPS cases. However, the databases used for this study would be unable to clearly differentiate among these cases due to a lack of access to actual ocular imaging data (e.g., FAF or OCT testing results).

Our study has some limitations. Selection bias or factors affecting the study patients may arise from the types of practices and healthcare providers who participate in the IRIS Registry, which are largely community practices. Thus, analyses may include limited representation from larger academic medical centers. In addition, exposure records to PPS would depend on availability of documentation in the claims database. Therefore, factors that preclude this documentation (e.g., no pharmacy claims for PPS) may result in these patients being excluded from the study population. Additionally, this study was limited in its ability to ascertain long-term PPS exposure, including any prior exposure period, given the earliest documentation date in the claims database was January 01, 2015. Also, if patients participated in patient assistance programs, PPS exposures might not be captured in the claims database.

Interpretation of study findings relies upon patients who could be linked to both the claims database and IRIS Registry. Therefore, study results may not be generalizable to patient populations or settings that are nonsimilar to clinical data sources used in this study, particularly to patients who had cumulative PPS exposure > 1000 grams, since our study included a very limited number of such patients (ie, 14 in the PPS overall cohort and none in the PPS clean cohort). Furthermore, this study may not be generalizable to populations exclusively visiting larger academic centers or those without insurance during the study period. Therefore, it is likely that the chances of detecting ophthalmologic disorders are greater than analyses based solely on claims records. Additionally, in the original case series by Pearce et. al describing pigmentary maculopathy in several patients receiving PPS, median length of exposure to PPS was 15.5 years [8]. This finding was also later replicated in a multi-center case series [12]. Finally, misclassification of retinal abnormality could be present, if imaging tests (OCT/FAF) were not performed.

In summary, our study suggested that the prevalence of PM/PR/Any was relatively common in both PPS-exposed cohorts (Clean and Overall) prior to PPS exposure. Crude incidence rates of PM/PR/Any varied slightly across the 3 cohorts but overall were relatively similar based on ITT analysis. Based on the study definition, incidence rate of PM/PR/PPS was relatively uncommon in both cohorts exposed to PPS, and a direct causal relationship could not be assessed.

## Supporting information

S1 Fig Selection flow for PPS Clean cohort. IC, interstitial cystitis; IRIS, Intelligent Research in Sight; PM, pigmentary maculopathy; PPS, pentosan polysulfate sodium; PR, pigmentary retinopathy; VA, visual acuity. ^a^Similar selection flow was used to identify eligible patients for the PPS Overall and Non-PPS-exposed IC cohorts. bDate of May 22, 2018 is referenced from the article by Pearce et al. (2018).
(PDF)

S1 Table Definition of study endpoint–PM/PR/Any. *Event date for the PM/PR/Any endpoint for a given patient is defined as the date of the earliest ICD-9/10 code documented from the above list’s corresponding ICD-9/10 codes; ICD, International Classification of Diseases; PM, pigmentary maculopathy; PR, pigmentary retinopathy.
(PDF)

S2 Table Baseline demographic and clinical characteristics for (A) PPS Overall and PPS Clean cohorts, and (B) Non-PPS-exposed IC cohort. (A) IQR, interquartile ratio; ITT, intent to treat; PM, pigmentary maculopathy; PPS, pentosan polysulfate sodium; PR, pigmentary retinopathy; SD, standard deviation. (B) IQR, interquartile ratio; ITT, intent to treat; PM, pigmentary maculopathy; PPS, pentosan polysulfate sodium; PR, pigmentary retinopathy; SD, standard deviation.
(PDF)

S3 Table Treating provider specialty for the PPS-exposed cohorts. IQR, interquartile range; N, number; PPS, pentosan polysulfate sodium; SD, standard deviation.
(PDF)

S4 Table PPS Clean cohort–Prevalence of PM/PR/Any stratified by age group, sex, race, and IC status. CI, confidence interval; IC, interstitial cystitis; N, number; PPS, pentosan polysulfate sodium; PM, pigmentary maculopathy; PR, pigmentary retinopathy.
(PDF)

S5 Table PPS Clean cohort–Incidence of PM/PR/Any stratified by age group, sex, race, and IC status. CI, confidence interval; IC, interstitial cystitis; ITT, intent to treat; N, number; OTT, on treatment time; PM, pigmentary maculopathy; PPS, pentosan polysulfate sodium; PR, pigmentary retinopathy.
(PDF)

S6 Table PPS Overall cohort–Prevalence of PM/PR/Any stratified by age group, sex, race, and IC status. CI, confidence interval; IC, interstitial cystitis; N, number; PPS, pentosan polysulfate sodium; PM, pigmentary maculopathy; PR, pigmentary retinopathy.
(PDF)

S7 Table PPS Overall cohort—Incidence of PM/PR/Any stratified by age group, sex, race, and IC status. CI, confidence interval; IC, interstitial cystitis; ITT, intent to treat; N, number; OTT, on treatment time; PPS, pentosan polysulfate sodium; PM, pigmentary maculopathy; PR, pigmentary retinopathy.
(PDF)

S8 Table Non-PPS-exposed IC cohort—Incidence of PM/PR/Any stratified by age group, sex, race, and IC status. CI, confidence interval; IC, interstitial cystitis; N, number; PPS, pentosan polysulfate sodium; PM, pigmentary maculopathy; PR, pigmentary retinopathy.
(PDF)

S9 Table VA changes among PPS-exposed patients. *<1 line of worsening or improvement considered not clinically meaningful; N, number; PM, pigmentary maculopathy; PPS, pentosan polysulfate sodium; PR, pigmentary retinopathy; VA, visual acuity.
(PDF)

S10 Table VA changes among Non-PPS-exposed IC patients. *<1 line of worsening or improvement considered not clinically meaningful; IC, interstitial cystitis; N, number; PM, pigmentary maculopathy; PPS, pentosan polysulfate sodium; PR, pigmentary retinopathy; VA, visual acuity.
(PDF)
