# Supplementary figures and images for: Retrospective study of incidence/prevalence of pigmentary maculopathy and retinopathy in patients receiving pentosan polysulfate sodium
Source: PLoS One. 2025 Jan 9;20(1):e0313497. doi: 10.1371/journal.pone.0313497 (PMC11717312; doi:10.1371/journal.pone.0313497)

**S1 Fig**

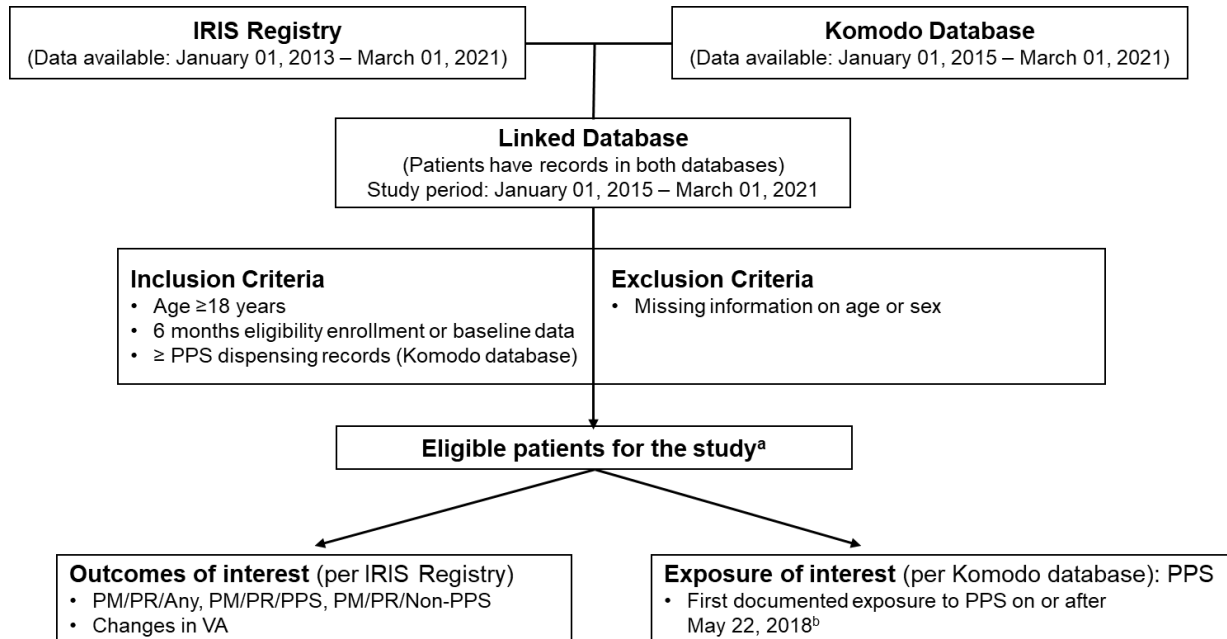

Supplement: S1 Fig — IC, interstitial cystitis; IRIS, Intelligent Research in Sight; PM, pigmentary maculopathy; PPS, pentosan polysulfate sodium; PR, pigmentary retinopathy; VA, visual acuity. aSimilar selection flow was used to identify eligible patients for the PPS Overall and Non-PPS-exposed IC cohorts. bDate of May 22, 2018 is referenced from the article by Pearce et al. (2018). (PDF) [file pone.0313497.s001.pdf]
