# Supplementary material for: Retrospective study of incidence/prevalence of pigmentary maculopathy and retinopathy in patients receiving pentosan polysulfate sodium
Source: PLoS One. 2025 Jan 9;20(1):e0313497. doi: 10.1371/journal.pone.0313497 (PMC11717312; doi:10.1371/journal.pone.0313497)
Supplement: S1 Table — *Event date for the PM/PR/Any endpoint for a given patient is defined as the date of the earliest ICD-9/10 code documented from the above list’s corresponding ICD-9/10 codes; ICD, International Classification of Diseases; PM, pigmentary maculopathy; PR, pigmentary retinopathy. (PDF) [file pone.0313497.s002.pdf]

S1 Table

Name	ICD 9	ICD 10
Toxic maculopathy	362.55	H35.38*
Hereditary retinal dystrophies (including hereditary maculopathy)	362.7*	H35.5*
Secondary pigmentary degeneration	362.65	H35.45*
Retinal dystrophy in other systemic disorders and syndromes	362.72	H36
Drusen	362.57	H35.36*
Nonexudative age-related macular degeneration	362.51	H35.31*
Exudative age-related macular degeneration	362.52	H35.32*
