# Supplementary material for: Retrospective study of incidence/prevalence of pigmentary maculopathy and retinopathy in patients receiving pentosan polysulfate sodium
Source: PLoS One. 2025 Jan 9;20(1):e0313497. doi: 10.1371/journal.pone.0313497 (PMC11717312; doi:10.1371/journal.pone.0313497)
Supplement: S2 Table — Baseline demographic and clinical characteristics for (A) PPS Overall and PPS Clean cohorts, and (B) Non-PPS-exposed IC cohort. (A) IQR, interquartile ratio; ITT, intent to treat; PM, pigmentary maculopathy; PPS, pentosan polysulfate sodium; PR, pigmentary retinopathy; SD, standard deviation. (B) IQR, interquartile ratio; ITT, intent to treat; PM, pigmentary maculopathy; PPS, pentosan polysulfate sodium; PR, pigmentary retinopathy; SD, standard deviation. (PDF) [file pone.0313497.s003.pdf]

S2 Table

A.

	PPS Overall Cohort		PPS Clean Cohort	
	**N (or mean/ median)**	**% [or SD/IQR]**	**N (or mean/ median)**	**% [or SD/IQR]**
**N (total)**	**14,053**	**100.00%**	**3,632**	**100.00%**
**Age, median (IQR)**	**57**	**[45, 67]**	**58**	**[44, 69]**
**Age, mean (SD)**	**55.91**	**15**	**56.01**	**17.26**
18-39, n (%)	2,360	16.79%	704	19.38%
40-59, n (%)	5,512	39.22%	1,258	34.64%
60-69, n (%)	3,363	23.93%	822	22.63%
>= 70, n (%)	2,818	20.05%	848	23.35%
**Sex, n (%)**				
Female	12,406	88.28%	3,153	86.81%
Male	1647	11.72%	479	13.19%
**Race, n (%)**				
White or Caucasian	9,814	69.84%	2,466	67.90%
Black or African American	661	4.70%	156	4.30%
Asian	179	1.27%	49	1.35%
Other	271	1.93%	68	1.87%
Unknown	3,128	22.26%	893	24.59%
**Ethnicity, n (%)**				
Hispanic	1056	7.51%	317	8.73%
Non-Hispanic	9,018	64.17%	2,200	60.57%
Unknown	3,979	28.31%	1,115	30.70%
**US Census Region, n (%)**				
Midwest: ND, SD, NE, KS, MO, MN, IA, WI, IL, IN, OH, MI	2,738	19.48%	682	18.78%
Northeast: ME, NH, VT, PA, NY, NJ, MA, RI, CT	3,261	23.21%	802	22.08%
South: MD, DE, WV, VA, DC, NC, SC, KY, TN, FL, GA, AL, MS, LA, AR, OK, TX	5,539	39.42%	1,417	39.01%
West: NM, AZ, CO, UT, WY, MT, ID, NV, CA, OR, WA, AK, HI	2,425	17.26%	707	19.47%
Unknown	90	0.64%	24	0.66%
**Payer Type, n (%)**				
Medicare	4,040	28.75%	975	26.84%
Medicaid	976	6.95%	307	8.45%
Military, Government	207	1.47%	52	1.43%
Commercial	6,209	44.18%	1,677	46.17%
No insurance	41	0.29%	12	0.33%
Miscellaneous	270	1.92%	91	2.51%
Unknown	2,310	16.44%	518	14.26%
**IC status**				
Had baseline IC	6,466	46.01%	2,111	58.12%
**Cumulative PPS dose (in categories)**				
· <500 (could be grouped with the category below, if# are low)	13,600	96.78%	3,630	99.94%
· 500 - <999 grams	439	3.12%	2	0.06%

	PPS Overall Cohort		PPS Clean Cohort	
	**N (or mean/ median)**	**% [or SD/IQR]**	**N (or mean/ median)**	**% [or SD/IQR]**
· 1000 –1500 grams	14	0.10%	0	0.00%
· >1500 grams	0	0.00%	0	0.00%
Cumulative PPS dose Median (IQR)	45.00	[18.00, 130.50]	27.00	[18.00, 54.00]
Cumulative PPS dose Mean (SD)	105.44	144.90	47.37	52.71
**General Comorbidities**				
Diabetes	2,503	17.81%	944	25.99%
Hypertension	3,671	26.12%	1,081	29.76%
Hypercholesterolemia	4,576	32.56%	2,010	55.34%
**Urological Comorbidities**				
Vaginitis	1467	10.44%	672	18.50%
UTI	5,922	42.14%	2,292	63.11%
Detrusor instability, urge incontinence, and overactive bladder	2,464	17.53%	1,059	29.16%
Radiation cystitis	168	1.20%	91	2.51%
**Autoimmune disease**				
Rheumatoid arthritis	583	4.15%	220	6.06%
Psoriasis/psoriatic arthritis	226	1.61%	86	2.37%
Systemic lupus erythematosus	214	1.52%	69	1.90%
Hashimoto’s thyroiditis	55	0.39%	14	0.39%
Graves’ disease	201	1.43%	119	3.28%
Sjogren syndrome	306	2.18%	120	3.30%
Myasthenia gravis	0	0.00%	0	0.00%
Inflammatory bowel disease (Crohn's disease, ulcerative colitis)	218	1.55%	92	2.53%
Vitiligo	22	0.16%	12	0.33%
Other autoimmune diseases	628	4.47%	286	7.87%
**Ocular Comorbidities (on or prior to index date)**				
Diabetic retinopathy	259	1.84%	107	2.95%
Diabetic macular edema	64	0.46%	23	0.63%
Optic neuropathy	0	0.00%	0	0.00%
Glaucoma (diagnosis)	1,508	10.73%	578	15.91%
Glaucoma-related procedure	146	1.04%	73	2.01%
Cataract (diagnosis)	3,654	26.00%	1,449	39.90%
Cataract (procedure)	751	5.34%	374	10.30%
Malignant tumor(s) of head and neck (plus documentation of radiation therapy)	0	0.00%	0	0.00%
**Ocular Comorbidities (after index date)**				
Diabetic retinopathy	340	2.42%	55	1.51%
Diabetic macular edema	129	0.92%	23	0.63%
Optic neuropathy	0	0.00%	0	0.00%
Glaucoma (diagnosis)	2,264	16.11%	368	10.13%
Glaucoma-related procedure	226	1.61%	28	0.77%
Cataract (diagnosis)	5,675	40.38%	793	21.83%
Cataract (procedure)	1,294	9.21%	129	3.55%
Malignant tumor(s) of head and neck (plus documentation of radiation therapy)	2	0.01%	0	0.00%

	PPS Overall Cohort		PPS Clean Cohort	
	N (or mean/ median)	% [or SD/IQR]	N (or mean/ median)	% [or SD/IQR]
**Ophthalmological Conditions Associated with Medication Use (any use on or prior to index date)**				
Pigmentary retinopathy	459	3.27%	166	4.57%
Optic neuropathy	2,617	18.62%	1,329	36.59%
Crystalline maculopathy	4,544	32.33%	1,903	52.40%
Cystoid Macular edema	112	0.80%	52	1.43%
**Ophthalmological Conditions Associated with Medication Use (any concomitant use after index date)**				
Pigmentary retinopathy	799	5.69%	154	4.24%
Optic neuropathy	4,762	33.89%	785	21.61%
Crystalline maculopathy	5,713	40.65%	1,112	30.62%
Cystoid Macular edema	180	1.28%	41	1.13%
**Ophthalmological Conditions Associated with Medication Use (concomitant use at any time during the study period)**				
Pigmentary retinopathy	934	6.65%	229	6.31%
Optic neuropathy	6,221	44.27%	1,658	45.65%
Crystalline maculopathy	7,742	55.09%	2,197	60.49%
Cystoid Macular edema	225	1.60%	68	1.87%
**Ophthalmological Conditions Associated with Medication Use (meeting cumulative exposure threshold on or prior to index date)**				
Pigmentary retinopathy	321	2.28%	135	3.72%
Optic neuropathy	183	1.30%	108	2.97%
Crystalline maculopathy	320	2.28%	168	4.63%
Cystoid Macular edema	59	0.42%	29	0.80%
**Ophthalmological Conditions Associated with Medication Use (meeting cumulative exposure threshold at any time during the study period)**				
Pigmentary retinopathy	753	5.36%	187	5.15%
Optic neuropathy	585	4.16%	150	4.13%
Crystalline maculopathy	1062	7.56%	273	7.52%
Cystoid Macular edema	171	1.22%	50	1.38%
**Treating Provider Specialty**				
Retina Specialist	1,461	10.40%	358	9.86%
Non-retina Specialist	4,190	29.82%	1,035	28.50%
General Ophthalmologist	3,847	27.37%	991	27.29%
Optometrist	3,887	27.66%	1,090	30.01%
Unknown	668	4.75%	158	4.35%
**Time from index date (months)**				
Study follow up time (ITT)[1]	40.82	22.12	17.25	9.35
On-treatment time[1]	26.41	23.13	14.29	9.97

B.

	IC cohort	
	N (or mean)	% (or SD)
**N (total)**	**48,546**	**100%**

	IC cohort	
	N (or mean)	% (or SD)
**Age, median (IQR)**	**59**	**[47, 69]**
**Age, mean (SD)**	**57**	**16**
18-39, n (%)	7,380	15.20%
40-59, n (%)	17,179	35.39%
60-69, n (%)	11,999	24.72%
>= 70, n (%)	11,988	24.69%
**Sex, n (%)**		
Female	43,685	89.99%
Male	4,861	10.01%
**Race, n (%)**		
White or Caucasian	34,123	70.29%
Black or African American	2,375	4.89%
Asian	579	1.19%
Other	891	1.84%
Unknown	10,578	21.79%
**Ethnicity, n (%)**		
Hispanic	3,671	7.56%
Non-Hispanic	31,236	64.34%
Unknown	13,639	28.10%
**US Census Region, n (%)**		
Midwest: ND, SD, NE, KS, MO, MN, IA, WI, IL, IN, OH, MI	12,594	25.94%
Northeast: ME, NH, VT, PA, NY, NJ, MA, RI, CT	10,946	22.55%
South: MD, DE, WV, VA, DC, NC, SC, KY, TN, FL, GA, AL, MS, LA, AR, OK, TX	15,601	32.14%
West: NM, AZ, CO, UT, WY, MT, ID, NV, CA, OR, WA, AK, HI	9,075	18.69%
Unknown	330	0.68%
**Payer Type, n (%)**		
Medicare	14,551	29.97%
Medicaid	3,082	6.35%
Military, Government	744	1.53%
Commercial	21,185	43.64%
No insurance	165	0.34%
Miscellaneous	1,055	2.17%
Unknown	7,764	15.99%
**General Comorbidities**		
Diabetes	11,181	23.03%
Hypertension	17,016	35.05%
Hypercholesterolemia	22,183	45.69%
**Urological Comorbidities**		
Vaginitis	6,613	13.62%
UTI	25,998	53.55%
Detrusor instability, urge incontinence, and overactive bladder	9,965	20.53%
Radiation cystitis	212	0.44%
**Autoimmune disease**		
Rheumatoid arthritis	2,308	4.75%
Psoriasis/psoriatic arthritis	1,012	2.08%
Systemic lupus erythematosus	862	1.78%
Hashimoto's thyroiditis	367	0.76%
Graves' disease	1,165	2.40%
Sjogren syndrome	1,301	2.68%
Myasthenia gravis	0	0.00%

	IC cohort	
	N (or mean)	% (or SD)
Inflammatory bowel disease (Crohn’s disease, ulcerative colitis)	858	1.77%
Vitiligo	125	0.26%
Other autoimmune diseases	2,997	6.17%
**Ocular Comorbidities (on or prior to index date)**		
Diabetic retinopathy	1,182	2.43%
Diabetic macular edema	402	0.83%
Optic neuropathy	2	0.00%
Glaucoma (diagnosis)	6,595	13.59%
Glaucoma-related procedure	632	1.30%
Cataract (diagnosis)	15,531	31.99%
Cataract (procedure)	3,233	6.66%
Malignant tumor(s) of head and neck (plus documentation of radiation therapy)	2	0.00%
**Ocular Comorbidities (after index date)**		
Diabetic retinopathy	1,162	2.39%
Diabetic macular edema	497	1.02%
Optic neuropathy	2	0.00%
Glaucoma (diagnosis)	7,593	15.64%
Glaucoma-related procedure	758	1.56%
Cataract (diagnosis)	18,079	37.24%
Cataract (procedure)	4,046	8.33%
Malignant tumor(s) of head and neck (plus documentation of radiation therapy)	4	0.01%
**Ophthalmological Conditions Associated with Medication Use (any use on or prior to index date)**		
Pigmentary retinopathy	1,428	2.94%
Optic neuropathy	8,542	17.60%
Crystalline maculopathy	14,303	29.46%
Cystoid macular edema	402	0.83%
**Ophthalmological Conditions Associated with Medication Use (any concomitant use after index date)**		
Pigmentary retinopathy	1,940	4.00%
Optic neuropathy	11,785	24.28%
Crystalline maculopathy	14,920	30.73%
Cystoid macular edema	470	0.97%
**Ophthalmological Conditions Associated with Medication Use (concomitant use at any time during the study period)**		
Pigmentary retinopathy	2,534	5.22%
Optic neuropathy	17,209	35.45%
Crystalline maculopathy	22,379	46.10%
Cystoid Macular edema	677	1.39%
**Ophthalmological Conditions Associated with Medication Use (meeting cumulative exposure threshold on or prior to index date)**		
Pigmentary retinopathy	1,066	2.20%
Optic neuropathy	584	1.20%
Crystalline maculopathy	895	1.84%
Cystoid macular edema	269	0.55%
**Ophthalmological Conditions Associated with Medication Use (meeting cumulative exposure threshold at any time during the study period)**		
Pigmentary retinopathy	1,929	3.97%

	IC cohort	
	**N (or mean)**	**% (or SD)**
Optic neuropathy	1,508	3.11%
Crystalline maculopathy	2,301	4.74%
Cystoid Macular edema	493	1.02%
**Treating Provider Specialty**		
Retina Specialist	4,777	9.84%
Non-retina Specialist	15,014	30.93%
General Ophthalmologist	13,178	27.15%
Optometrist	13,115	27.02%
Unknown	2,462	5.07%
**Time from Index Date, months**		
Study follow up time (ITT)[1]	35.49	21.54
