# Supplementary material for: Retrospective study of incidence/prevalence of pigmentary maculopathy and retinopathy in patients receiving pentosan polysulfate sodium
Source: PLoS One. 2025 Jan 9;20(1):e0313497. doi: 10.1371/journal.pone.0313497 (PMC11717312; doi:10.1371/journal.pone.0313497)
Supplement: S3 Table — IQR, interquartile range; N, number; PPS, pentosan polysulfate sodium; SD, standard deviation. (PDF) [file pone.0313497.s004.pdf]

S3 Table

	PPS Overall Cohort		PPS Clean Cohort	
	N (or mean/ median)	% (or SD/IQR)	N (or mean/ median)	% (or SD/IQR)
N (total)	14,053	100.00%	3,632	100.00%
Retina Specialist	1,461	10.40%	358	9.86%
Non-retina Specialist	4,190	29.82%	1,035	28.50%
General Ophthalmologist	3,847	27.37%	991	27.29%
Optometrist	3,887	27.66%	1,090	30.01%
Unknown	668	4.75%	158	4.35%
