# Supplementary material for: Retrospective study of incidence/prevalence of pigmentary maculopathy and retinopathy in patients receiving pentosan polysulfate sodium
Source: PLoS One. 2025 Jan 9;20(1):e0313497. doi: 10.1371/journal.pone.0313497 (PMC11717312; doi:10.1371/journal.pone.0313497)
Supplement: S4 Table — CI, confidence interval; IC, interstitial cystitis; N, number; PPS, pentosan polysulfate sodium; PM, pigmentary maculopathy; PR, pigmentary retinopathy. (PDF) [file pone.0313497.s005.pdf]

S4 Table

		N=3,632	
**Stratification**	**Total**	**Count of patients with endpoint**	**Prevalence rate (95% CI)**
**Age**			
Ages 18-39	704	8	1.14 (0.35, 1.92)
Ages 40-59	1,258	42	3.34 (2.35, 4.33)
Ages 60-69	822	67	8.15 (6.28, 10.02)
Ages ≥70	848	189	22.29 (19.49, 25.09)
**Sex**			
Female	3,153	250	7.93 (6.99, 8.87)
Male	479	56	11.69 (8.81, 14.57)
**Race**			
White or Caucasian	2,466	229	9.29 (8.14, 10.43)
Black or African American	156	14	8.97 (4.49, 13.46)
Asian	49	3	6.12 (0.0, 12.84)
Other	68	3	4.41 (0.0, 9.29)
Unknown	893	57	6.38 (4.78, 7.99)
**IC Status**			
Baseline IC	2,111	160	7.58 (6.45, 8.71)
No Baseline IC	1,521	146	9.60 (8.12, 11.08)
