# Supplementary material for: Retrospective study of incidence/prevalence of pigmentary maculopathy and retinopathy in patients receiving pentosan polysulfate sodium
Source: PLoS One. 2025 Jan 9;20(1):e0313497. doi: 10.1371/journal.pone.0313497 (PMC11717312; doi:10.1371/journal.pone.0313497)
Supplement: S5 Table — CI, confidence interval; IC, interstitial cystitis; ITT, intent to treat; N, number; OTT, on treatment time; PM, pigmentary maculopathy; PPS, pentosan polysulfate sodium; PR, pigmentary retinopathy. (PDF) [file pone.0313497.s006.pdf]

S5 Table

	N=3,331							
Stratification	Total persons at risk	Count of patients with endpoint ITT	Incident proportion (95% CI)	Count of patients with endpoint	Total OTT at risk (per 100 person-years)	(95% CI)	Total ITT time at risk (per 100 person-year	(95% CI)
**Age**								
Ages 18-39	696	2	0.29 (0.11, 0.69)	1	0.12	0.20	(0.00, 0.37)	(0.0, 0.48)
Ages 40-59	1,217	24	1.97 (1.19, 2.75)	18	1.23	1.34	(0.66, 1.79)	(0.81, 1.88)
Ages 60-69	758	41	5.41 (3.80, 7.02)	36	3.89	3.68	(2.62, 5.15)	(2.56, 4.81)
Ages ≥70	660	35	5.30 (3.59, 7.01)	27	3.57	3.91	(2.22, 4.91)	(2.61, 5.21)
**Sex**								
Female	2,907	93	3.20 (2.56, 3.84)	73	2.11	2.22	(1.63, 2.60)	(1.77, 2.67)
Male	424	9	2.12 (0.75, 3.49)	9	1.78	1.52	(0.62, 2.95)	(0.53, 2.52)
**Race**								
White or Caucasian	2,241	74	3.30 (2.56, 4.04)	61	2.27	2.29	(1.70, 2.84)	(1.77, 2.81)
Black or African American	142	3	2.11 (0.0, 4.48)	1	0.56	1.36	(0.00, 1.66)	(0.0, 2.89)
Asian	46	2	4.35 (0.0, 10.24)	2	3.32	3.13	(0.00, 7.93)	(0.0, 7.46)
Other	65	5	7.69 (1.21, 14.17)	3	4.35	5.73	(0.00, 9.27)	(0.71, 10.75)
Unknown	837	18	2.15 (1.17, 3.13)	15	1.56	1.54	(0.77, 2.35)	(0.83, 2.25)
**IC Status**								
Baseline IC	1,954	57	2.92 (2.17, 3.66)	43	1.9	2.03	(1.33, 2.46)	(1.50, 2.55)

N=3,331								
**Stratification**	**Total persons at risk**	**Count of patients with endpoin t ITT**	**Incident proportion (95% CI)**	**Count of patients with endpoint**	**Total OTT at risk (per 100 person-years)**	**(95% CI)**	**Total ITT time at risk (per 100 person-year**	**(95% CI)**
No Baseline IC	1,377	45	3.27 (2.33, 4.21)	39	2.31	2.29	(1.58, 3.03)	(1.62, 2.96)
