# Supplementary material for: Retrospective study of incidence/prevalence of pigmentary maculopathy and retinopathy in patients receiving pentosan polysulfate sodium
Source: PLoS One. 2025 Jan 9;20(1):e0313497. doi: 10.1371/journal.pone.0313497 (PMC11717312; doi:10.1371/journal.pone.0313497)
Supplement: S6 Table — CI, confidence interval; IC, interstitial cystitis; N, number; PPS, pentosan polysulfate sodium; PM, pigmentary maculopathy; PR, pigmentary retinopathy. (PDF) [file pone.0313497.s007.pdf]

S6 Table

	N = 4,053		
**Stratification**	**Total**	**Count of patients with endpoint**	**Prevalent rate (95% CI)**
**Age**			
Ages 18-39	2,360	13	0.55 (0.25, 0.85)
Ages 40-59	5,512	72	1.31 (1.01, 1.61)
Ages 60-69	3,363	136	4.04 (3.38, 4.71)
Ages ≥70	2,818	363	12.88 (11.64, 14.12)
**Sex**			
Female	12,406	485	3.91 (3.57, 4.25)
Male	1,647	99	6.01 (4.86, 7.16)
**Race**			
White or Caucasian	9,814	458	4.67 (4.25, 5.08)
Black or African American	661	20	3.03 (1.72, 4.33)
Asian	179	9	5.03 (1.83, 8.23)
Other	271	11	4.06 (1.71, 6.41)
Unknown	3,128	86	2.75 (2.18, 3.32)
**IC Status**			
Baseline IC	6,466	295	4.56 (4.05, 5.07)
No Baseline IC	7,587	289	3.81 (3.38, 4.24)
