# Supplementary material for: Retrospective study of incidence/prevalence of pigmentary maculopathy and retinopathy in patients receiving pentosan polysulfate sodium
Source: PLoS One. 2025 Jan 9;20(1):e0313497. doi: 10.1371/journal.pone.0313497 (PMC11717312; doi:10.1371/journal.pone.0313497)
Supplement: S7 Table — CI, confidence interval; IC, interstitial cystitis; ITT, intent to treat; N, number; OTT, on treatment time; PPS, pentosan polysulfate sodium; PM, pigmentary maculopathy; PR, pigmentary retinopathy. (PDF) [file pone.0313497.s008.pdf]

S7 Table

				N=13,486				
**Stratification**	**Total persons at risk**	**Count of patients with endpoint**	**Incident proportion (95% CI)**	**Count of patients with endpoint for OTT analysis**	**Incidence Rate OTT time at risk (per 100 person-years)**	**Incidence Rate ITT time at risk (per 100 person-years)**	**Incidence Rate 95% CI [OTT time-at-risk]**	**Incidence Rate 95% CI [ITT time-at-risk]**
**Age**								
Ages 18-39	2,347	28	1.19 (0.75, 1.63)	18	0.36	0.38	(0.19, 0.52)	(0.24, 0.52)
Ages 40-59	5,442	343	6.30 (5.66, 6.95)	201	1.64	1.74	(1.42, 1.87)	(1.56, 1.93)
Ages 60-69	3,236	419	12.95 (11.79, 14.10)	280	3.79	3.71	(3.35, 4.24)	(3.36, 4.07)
Ages ≥70	2,461	498	20.24 (18.65, 21.82)	339	6.8	6.67	(6.07, 7.52)	(6.08, 7.25)
**Sex**								
Female	11,935	1173	9.83 (9.29, 10.36)	745	2.85	2.88	(2.65, 3.06)	(2.71, 3.04)
Male	1,551	115	7.41 (6.11, 8.72)	93	2.64	2.27	(2.10, 3.18)	(1.86, 2.69)
**Race**								
White or Caucasian	9,371	1015	10.83 (10.20, 11.46)	665	3.21	3.16	(2.97, 3.45)	(2.96, 3.35)
Black or African American	641	32	4.99 (3.31, 6.68)	20	1.41	1.42	(0.79, 2.03)	(0.93, 1.91)
Asian	170	12	7.06 (3.21, 10.91)	6	1.5	2.09	(0.30, 2.70)	(0.91, 3.27)
Other	261	26	9.96 (6.33, 13.60)	20	3.54	3.12	(1.99, 5.09)	(1.92, 4.32)
Unknown	3,043	203	6.67 (5.78,	127	1.94	2.03	(1.60,	(1.75, 2.31)

Stratification	Total persons at risk	Count of patients with endpoint	Incident proportion (95% CI)	Count of patients with endpoint for OTT analysis	Incidence Rate OTT time at risk (per 100 person-years)	Incidence Rate ITT time at risk (per 100 person-years)	Incidence Rate 95% CI [OTT time-at-risk]	Incidence Rate 95% CI [ITT time-at-risk]
N=13,486								
			7.56)				2.28)	
**IC Status**								
Baseline IC	6,180	436	7.06 (6.42, 7.69)	285	2.25	2.32	(1.98, 2.51)	(2.10, 2.54)
No Baseline IC	7,306	852	11.66 (10.93, 12.40)	553	3.26	3.15	(2.99, 3.53)	(2.94, 3.36)
