# Supplementary material for: Retrospective study of incidence/prevalence of pigmentary maculopathy and retinopathy in patients receiving pentosan polysulfate sodium
Source: PLoS One. 2025 Jan 9;20(1):e0313497. doi: 10.1371/journal.pone.0313497 (PMC11717312; doi:10.1371/journal.pone.0313497)
Supplement: S8 Table — CI, confidence interval; IC, interstitial cystitis; N, number; PPS, pentosan polysulfate sodium; PM, pigmentary maculopathy; PR, pigmentary retinopathy. (PDF) [file pone.0313497.s009.pdf]

S8 Table

		N = 45,930			
Stratification	Total persons at risk	Count of patients with endpoint	Incident proportion (95% CI)	Total ITT time at risk (per 100 person-years)	Incident (95% CI) (ITT) time-at-risk
**Age**					
Ages 18-39	7,338	53	0.72 (0.53, 0.92)	0.27	(0.20, 0.34)
Ages 40-59	16,886	535	3.17 (2.90, 3.43)	1.02	(0.93, 1.10)
Ages 60-69	11,404	904	7.93 (7.43, 8.42)	2.58	(2.41, 2.75)
Ages ≥70	10,302	1,736	16.85 (16.13, 17.57)	6.11	(5.83, 6.40)
**Sex**					
Female	41,391	2,857	6.90 (6.66, 7.15)	2.33	(2.25, 2.42)
Male	4,539	371	8.17 (7.38, 8.97)	2.84	(2.55, 3.13)
**Race**					
White or Caucasian	32,026	2549	7.96 (7.66, 8.26)	2.66	(2.56, 2.76)
Black or African American	2,286	96	4.20 (3.38, 5.02)	1.44	(1.15, 1.72)
Asian	550	36	6.55 (4.48, 8.61)	2.3	(1.55, 3.05)
Other	853	50	5.86 (4.29, 7.44)	2.06	(1.49, 2.63)
Unknown	10,215	497	4.87 (4.45, 5.28)	1.71	(1.56, 1.86)
