# Supplementary material for: Retrospective study of incidence/prevalence of pigmentary maculopathy and retinopathy in patients receiving pentosan polysulfate sodium
Source: PLoS One. 2025 Jan 9;20(1):e0313497. doi: 10.1371/journal.pone.0313497 (PMC11717312; doi:10.1371/journal.pone.0313497)
Supplement: S9 Table — *<1 line of worsening or improvement considered not clinically meaningful; N, number; PM, pigmentary maculopathy; PPS, pentosan polysulfate sodium; PR, pigmentary retinopathy; VA, visual acuity. (PDF) [file pone.0313497.s010.pdf]

S9 Table

		PPS clean cohort			PPS overall cohort		
	**VA changes from baseline**	**Number of patients with VA changes**	**% of patients with VA changes**	**Subgroup N**	**Number of patients with VA changes**	**% of patients with VA changes**	**Subgroup N**
**Without PM/PR/Any during the follow-up period**	≥3 lines of worsening	31	4.55%	681	278	4.68%	5935
	1 to <3 lines of worsening	150	22.03%		1490	25.11%	
	No change*	347	50.95%		2786	46.94%	
	1 to <3 lines of improvement	123	18.06%		1143	19.26%	
	≥3 lines of improvement	30	4.41%		238	4.01%	
**With PM/PR/Any during the follow-up period**	≥3 lines of worsening	13	9.03%	144	135	12.00%	1125
	1 to <3 lines of worsening	44	30.56%		329	29.24%	
	No change*	54	37.50%		385	34.22%	
	1 to <3 lines of improvement	28	19.44%		233	20.71%	
	≥3 lines of improvement	5	3.47%		43	3.82%	
**PM/PR/PPS**	≥3 lines of worsening	1	20.00%	5	6	10.00%	59
	1 to <3 lines of worsening	1	20.00%		20	33.90%	
	No change*	1	20.00%		21	35.59%	
	1 to <3 lines of improvement	1	20.00%		11	18.64%	
	≥3 lines of improvement	1	20.00%		1	2.00%	
