# Supplementary material for: Retrospective study of incidence/prevalence of pigmentary maculopathy and retinopathy in patients receiving pentosan polysulfate sodium
Source: PLoS One. 2025 Jan 9;20(1):e0313497. doi: 10.1371/journal.pone.0313497 (PMC11717312; doi:10.1371/journal.pone.0313497)
Supplement: S10 Table — *<1 line of worsening or improvement considered not clinically meaningful; IC, interstitial cystitis; N, number; PM, pigmentary maculopathy; PPS, pentosan polysulfate sodium; PR, pigmentary retinopathy; VA, visual acuity. (PDF) [file pone.0313497.s011.pdf]

S10 Table

	VA changes from baseline	Number of patients with VA changes	% of patients with VA changes
**Without PM/PR/Any during the follow-up period**	≥3 lines of worsening	842	4.59%
	1 to <3 lines of worsening	4498	24.51%
	No change*	8837	48.16%
	1 to <3 lines of improvement	3484	18.99%
	≥3 lines of improvement	689	3.75%
**With PM/PR/Any during the follow-up period**	≥3 lines of worsening	335	10.30%
	1 to <3 lines of worsening	994	30.57%
	No change*	1168	35.92%
	1 to <3 lines of improvement	628	19.31%
	≥3 lines of improvement	127	3.91%
